# Blood Circulating Non-Coding RNAs for the Clinical Management of Triple-Negative Breast Cancer

**DOI:** 10.3390/cancers14030803

**Published:** 2022-02-04

**Authors:** Tomasz Powrózek, Michael Ochieng Otieno

**Affiliations:** 1 Department of Human Physiology, Medical University of Lublin, 20-080 Lublin, Poland; 2 Haematological Malignancies H12O Clinical Research Unit, Spanish National Cancer Research Centre, 28029 Madrid, Spain; michaelochieng@yahoo.com

**Keywords:** triple negative breast cancer, ncRNAs, liquid biopsy, biomarker, bioinformatics

## Abstract

**Simple Summary:**

Nowadays, in clinics, there is a lack of reliable biomarkers that could serve as tools allowing for early cancer detection, prediction of therapy response, tumor recurrence, and TNBC course. In this review, we summarized the most recent findings on the applicability of unique blood circulating ncRNAs for management of TNBC. This review was supplemented by bioinformatics analysis for better understanding of molecular processes in which ncRNAs are involved, to promote individual TNBC phenotype and tumor action.

**Abstract:**

Triple negative breast cancer (TNBC) represents the most aggressive subtype of breast cancer, and is related to unfavorable prognosis and limited treatment strategies. Currently, there is a lack of reliable biomarkers allowing for the clinical management of TNBC. This is probably caused by a complex molecular background, leading to the development and establishment of a unique tumor phenotype. Recent studies have reported non-coding RNAs (ncRNAs) not only as the most promising class of molecular agents with a high applicability to manage human cancers, including TNBC, but also as robust and non-invasive biomarkers that are able to be monitored in blood circulation, with the application of liquid biopsy. There is a lack of papers discussing the role of blood-circulating ncRNAs as diagnostic, predictive, and prognostic biomarkers for TNBC. In this paper, we summarized the available literature reports on the utility of blood-circulating ncRNAs for TNBC management. Additionally, we supplemented this review by bioinformatics analysis, for better understanding of the role of ncRNAs’ machinery in the development of a unique TNBC phenotype.

## 1. Introduction

### 1.1. Triple-Negative Breast Cancer

Despite the advances in diagnosis and implementation of adequate treatment options, including tailored targeted therapies, breast cancer (BC) is the second most common cause of cancer-related deaths in women worldwide [1,2]. The recent efforts made toward the improvement of treatment strategies achieved a decrease in BC mortality of about 3% and progress in the 5 year survival rate up of to 80%, depending on cancer subtype and disease stage [3,4]. Currently, more younger patients are unfortunately diagnosed with the presence of either local or metastatic disease, and in spite of applied treatment, the majority of them will eventually develop distant metastases and/or tumor recurrence [5,6]. It is probably caused by presence of more aggressive subtypes of BC in this group of patients.

Among the BC histological subtypes, the triple-negative breast cancer (TNBC) differs from others by its substantial aggressiveness, limited therapy options, and the poorest prognosis [7,8]. It is often diagnosed in women aged <40 years and in an advanced stage of the disease, with the corresponding presence of metastases into distant organs [9]. Management of TNBC is still challenging, due to its high clinical and molecular heterogeneity that significantly differs from other BC subtypes. Moreover, the development of both drug resistance and progressive disease limit the therapy perspectives for incomplete responders and recurrent patients [10]. As mentioned above, TNBC is more often associated with hereditary conditions when compared to other BC subtypes, caused by considerable genetic heterogeneity [10,11]. Indeed, several highly effective approaches including genomics, transcriptomics, and epigenetics have revealed substantial heterogeneity within TNBC, with it having sets of molecular alterations unique for this BC subtype [12,13,14]. It is hypothesized that this exceptional molecular pattern is a result of the coexistence of penetrating genetic alterations, contributing to both the clinical differences and enhanced aggressiveness of the TNBC phenotype [15]. Because of the molecular complexity affecting tumor behavior, TNBC remains unpredictable and adds some difficulty in the recent attempts to improve strategies for disease control [16].

Nowadays, in clinics, there is a lack of reliable biomarkers that could serve as tools allowing for early cancer detection, prediction of therapy response, tumor recurrence, and TNBC course. Recent efforts made to adapt novel techniques to reveal such biomarkers have proven futile to some extent. Hitherto, clinical decisions are made exclusively upon either histopathologic analysis or analysis of a small number of genes, including their coding proteins in the tumor tissue, which are also distinctly limited [15,17,18]. Moreover, the widespread use of high-throughput profiling techniques or using the commercially available genetic signatures is also of limited application, mainly due to their cost and reproducibility issues [19]. The recent studies have reported non-coding RNAs (ncRNAs) as the most promising class of molecular agents with a high applicability to manage human cancers, including BC, as a robust and non-invasive biomarker that can be monitored in blood circulation [20]. Moreover, a novel TNBC subtyping system, assigning TNBC patients to four distinct subtypes by integrating both mRNA and lncRNA expression profiles, was also proposed [21]. In this review, we summarized the most recent findings on the applicability of unique blood circulating ncRNAs for detection, prediction, and prognosis of TNBC. The review was supplemented by bioinformatics analysis for better understanding of molecular processes in which ncRNAs are involved, to promote individual TNBC phenotype and tumor action.

### 1.2. ncRNAs

The majority of transcripts in the human genome are non-coding sequences, that represent a regulatory role in the whole molecular processes of the cell. ncRNAs represent the largest family of RNAs that are not coding for proteins, and form a significant proportion of the genome. According to the recent findings, the three of the most important players in the regulation of gene expression were identified in the ncRNAs family, as followed by microRNA (miRNA), long non-coding RNA (lncRNA), and circular RNA (circRNA) [22,23]. Their expression profiles can be used to discriminate between healthy and neoplastic states, as well as between different types of cancer [22,24]. Until now, the miRNAs are the most studied group of ncRNAs, and numerous papers provide their high applicability in clinics for tumor detection, prediction, and prognosis [24]. Briefly, miRNAs can mediate gene regulation by post-transcriptionally binding to the 3′ untranslated region (3′-UTR) of their target mRNA, acting as oncogenes or tumor suppressors. In the case of miRNAs deregulation, gene expression either accelerates or undergoes silencing, affecting the protein level [25,26]. There are two reasons why miRNAs are involved in complex molecular networks. On the one hand, the sequence of one miRNA can target multiple mRNAs. On the other hand, a single mRNA can be targeted by multiple miRNAs [27]. Perhaps, this complexity and low tumor specificity are a major disadvantage for miRNAs as an ideal cancer biomarker. Nevertheless, miRNAs seem to accurately mediate the phenotype of TNBC by regulation of tumor aggressiveness, migration, proliferation, and invasiveness [17,28]. Moreover, it was also proven that miRNAs can mediate the response of TNBC to chemotherapy and isan attractive target for future anti-miRNA therapy [29]. In contrast to miRNAs, lncRNAs were found to be involved in transcriptional and post-transcriptional genome regulation, through interactions with DNA, RNA, and proteins. lncRNAs can either promote or inhibit the formation of transcription loops to regulate gene transcription. Besides, lncRNAs also regulate mRNA splicing and act as precursors to other ncRNAs, such as miRNAs. The function of lncRNA as tumor suppressors or oncogenes was noticed in different cellular signaling pathways [30,31]. Similar to miRNAs, these molecules play crucial role in carcinogenesis, and some of them were identified as crucial for TNBC progression, such as MALAT1, HOTAIR, ANRILA, or NEF [32]. The most recently discovered and still not fully recognized group of ncRNAs are circRNAs. They represent a covalently closed, continuous loop of structures, and the 3′ and 5′ ends have been joined together (create circular forms), which differs them from other known ncRNAs. In contrast to the canonical splicing of mRNAs, circRNAs originate from spliceosome-mediated, non-sequential back-splicing of pre-mRNAs. Thanks to their structure, circRNAs are protected from degradation by RNase or RNA exonucleases, which makes circRNAs more stable than other ncRNAs, and their half-life is about five times longer than that of mRNA. In contrast to other ncRNAs, the mechanism participating in circRNAs’ degradation is still not fully understood, however, it is believed that the putative role in their global degradation is played by RNase L. While most of circRNAs have not been identified yet, and their particular role of known sequences is unclear, they demonstrate a putatively important role in the regulation of genomic machinery. It is widely accepted that circRNAs are significant regulators that influence both physiological and pathological conditions by regulating splicing mechanisms, acting as sponges for different miRNAs, and regulating epigenetic alterations (DNA and histone methylation). In transcriptional regulation, circRNAs can also regulate protein functions by forming complexes with proteins and alter their function and expression, suggesting their role in carcinogenesis and the stemness of cancer. circRNAs play an important role in tumor progression by modulating the hallmarks of cancer, mainly by the regulation of sustained proliferative signaling, the eluding of growth suppressors, and the impairment of differentiation signals. circRNAs promote tumor metastasis and invasion and induce angiogenesis [33,34,35]. Truly, some circRNAs were recently found in solid tumors participating in tumor progression and cell invasiveness. In TNBC, the following circRNAs were identified as tumor suppressors or oncogenes: circKIF4A, circITCH, circMTO1, circAGFG1, circSEPT9, and others [36]. Currently, it is believed that understanding of simultaneous interactions between the group of discussed ncRNAs can broaden the knowledge on carcinogenesis. However, the exact function and mechanism of action of most of them is still unknown. ncRNAs create a very complex network of mutual interactions and act as oncogenes or tumor suppressors. These events are unique for cancer states and are as a result of dual interaction between biological and pathological processes in the body (host–tumor interaction). ncRNAs demonstrate a tissue-specific expression pattern, which is highly altered in cancer, and are considered to be promising diagnostic, prognostic, and therapeutic targets.

**Figure 1:**
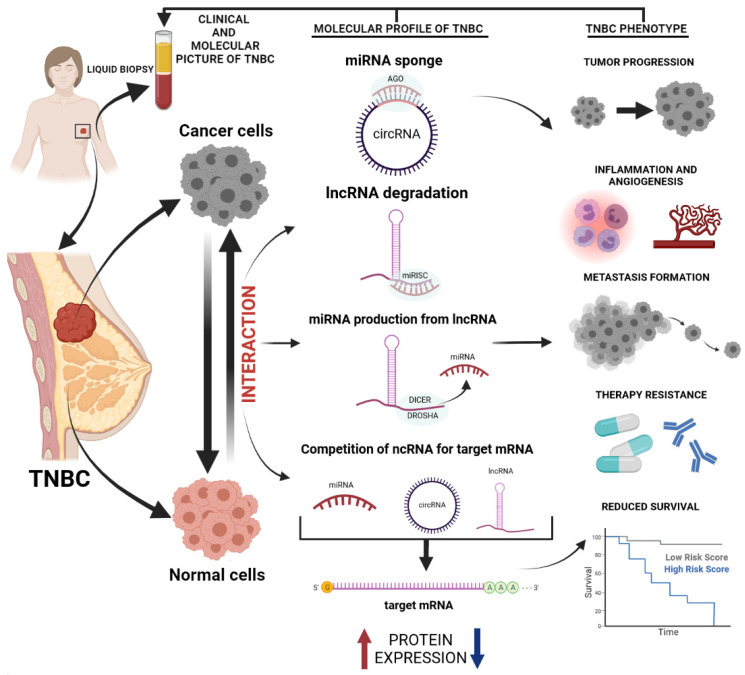
Interaction between the tumor and the host resulting in alteration of ncRNAs machinery for the development of the unique phenotype of TNBC and the usefulness of liquid biopsy for the tumor management—altered expression pattern of ncRNAs drivesan unfavorable phenotype of cancer, therefore profiling of ncRNAs in blood sample reflects molecular and clinical picture of TNBC.

One of the major pros for the analysis of ncRNAs as TNBC biomarkers is their remarkable stability in body fluids and significantly altered expression under the cancer conditions. They are both released into blood circulation from cancerous tissues (directly or within the exosomes) or from host tissues affected by tumor occurrence [37]. Moreover, the monitoring of ncRNAs’ expression with the use of liquid biopsy, especially in blood circulation (plasma or serum), is more convenient, cheaper, and safer for patients than a series of tissue biopsies. It also allows to capture the entire heterogeneity of the tumor (primary/metastatic/recurrent) in a minimally invasive manner. The additional predominance of liquid biopsy over other techniques is that it may allow for the stratification and real-time monitoring of therapies. Liquid biopsy can provide identification of therapeutic targets and can be easily repeated if needed, and can be used as often as necessary to monitor the patient’s progress. It is worth noting that liquid biopsy is not free from limitations. The tissue biopsy is still a diagnostic “gold standard” for cancer, because it provides a significantly higher yield of cancer sample (cancer cells, nucleic acids) than liquid biopsy. The accuracy and sensitivity challenges of liquid biopsy still exist, because nucleic acids are relatively rare in blood circulation. Eventually, it is still not clear if this technique provides a representative sampling of all genetic alterations of cancer cells or if there is a bias to specific sub-regions of the tumor. Liquid biopsy as a diagnostic technique still requires further clinical validation [38,39]. Nevertheless, the idea of the introduction of liquid biopsy for cancer management was already confirmed by some clinical papers, including in BC control [39]. Nevertheless, there are still limited data concerning the utility of circulating ncRNAs exclusively for TNBC management. In Figure 1, we illustrated the concept of host–tumor interactions, resulting in alteration of ncRNAs’ expression and their putative impact on TNBC course. The developing tumor forms a unique microenvironment affecting both cancer and normal cells, leading to their interaction (tumor–host interaction). Cancer cells, by changing fibroblasts’ behavior, macrophage secretion, and exosome synthesis, enhance tumor growth, proliferation, and invasiveness. Under the influence of tumor environment, healthy cells disintegrate, change their metabolism, and develop an inflammatory response. Tumor–host interaction results in the alteration of nucleic acid expression, including a series of alterations exclusive for ncRNAs (changes in miRNA synthesis and sponging by circRNAs, lncRNAs degradation, and competition of ncRNAs for targeted mRNA). Following changes of protein expression, they participate in the creation of a unique phenotype of cancer, characterized by a various capacity to progression, grade of inflammation, and therapy outcomes.

## 2. Circulating ncRNAs for TNBC Detection

We conducted a literature search until October, 2021 using the databases: PubMed, EMBASE, Scopus, and by manual searching with the use of keywords (Google) for papers written in the English language. The following approach of literature searching was used with the application of keywords: “triple-negative breast cancer”, “TNBC”, “breast cancer”, “blood”, “serum”, “plasma”, then supplemented by “miRNA”, “microRNA”, “miR”, “long non-coding RNA”, “lncRNA”, “circular RNA”, and “circRNA”. Also, the following search builder was used: (“triple-negative breast cancer” OR “TNBC” OR “breast cancer”) AND (“blood” OR “serum” OR “plasma”) AND (“miRNA” OR “microRNA” OR “miR) AND (“long non-coding RNA” OR “lncRNA”) AND “circular RNA” OR “circRNA”). In Table 1, we summarized findings on circulating ncRNAs for non-invasive diagnosis of TNBC [40,41,42,43,44,45,46,47,48,49,50,51,52,53,54,55,56,57,58,59,60,61,62,63,64,65,66,67,68,69,70,71,72,73,74,75,76,77]. Additionally, examples of validated targets and for the putative role in TNBC carcinogenesis were provided for all discussed ncRNAs.

### 2.1. miRNAs

Notably, circulating miRNAs were widely examined as prospective diagnostic biomarkers of human malignancies, including BC. However, only a few papers focused on their diagnostic accuracy exclusively for TNBC. In Table 1, we summarized all studies on the utility of blood circulating miRNAs for TNBC diagnosis [40,41,42,43,44,45,46,47,48,49,50,51,52,53,54,55,56,57,58,59,60,61,62,63,64,65].

**Figure 2:**
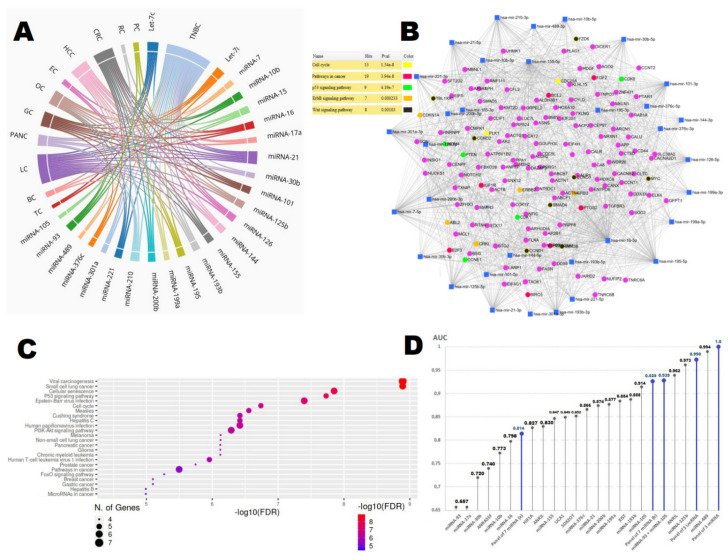
Bioinformatics analysis of the blood circulating miRNAs and lncRNAs serving as diagnostic biomarkers of TNBC: (**A**)—chord diagram demonstrating the relationship between circulating miRNAs found in TNBC and their relationship to different cancers; (**B**)—regulatory network of the studied miRNAs, followed by KEGG enrichment analysis (genes crucial for the unique pathways were marked by appropriate colors); (**C**)—KEGG enrichment analysis for the circulating lncRNAs; (**D**)—diagnostic accuracy of the discussed ncRNAs for the non-invasive detection of TNBC; (BC—breast cancer, CRC—colorectal cancer, EC—esophageal cancer, GC—gastric cancer, HCC—hepatocellular cancer, LC—lung cancer, OC—ovarian cancer, PANC—pancreatic cancer, PC—prostate cancer, RC—renal cancer, TC—thyroid cancer).

One of the first papers that has begun a series of works analyzing the utility of miRNAs for TNBC detection was the study of Shin et al. Using microarray (MA) followed by qRT-PCR validation, they selected miRNA-16,21 and 199a as promising TNBC biomarkers, and achieved quite satisfactory diagnostic accuracy for TNBC detection with the AUC ranging from 0.798 to 0.884. Interestingly, authors also found that expression of studied miRNAs differs between pre- and post-operative patients, suggesting miRNAs as non-invasive markers for TNBC monitoring [40]. Some of the circulating miRNAs summarized in Table 1 confirmed their clinical value by reflecting tumor stage, lymph node status, and metastases occurrence. For instance, miRNA-21, 199a, 210, and 221 were found to be associated with tumor stage, whereas expression of miRNA-10b, 17a, 30b, 93, 105, and 376c correlated with lymph node status and the presence of distant metastases [40,42,48,61,65]. Among the studied miRNAs, few molecules were identified as being significant for TNBC in independent studies, such as miRNA-21, miRNA-199a, and 489 [40,42,43,57]. It is still debatable whether single-circulating miRNA can serve as an objective and reliable biomarker of cancer so far. Visibly, most of the analyzed single-circulating miRNA demonstrated high diagnostic accuracy for TNBC, which is proven by high AUC values: miRNA-489 (0.994), 125b (0.973), 105 (0.928), and 193b (0.914). On the contrary, there are some miRNAs that suffer from low diagnostic accuracy: miRNA-16 (0.657), 17a (0.657), and 30b (0.720) [48,57,61,65]. According to most researchers, the high diagnostic reliability can be achieved only by a combination of some miRNAs into diagnostic signatures. Truly, higher accuracy and reliability was noted for TNBC in the case of combination of two, three or seven miRNAs with the following AUCs: 0.939, 1.0 and 0.929, respectively [42,43,48]. Moreover, in some papers it has been proven that expression of blood miRNAs correlates with their expression in cancer tissue, for instance, with miRNA-16, 21, 199a [40], 210, 221 [42], and 200 [57]. Those finding suggest that blood miRNAs are a reflection of their tissue expression, which can allow analysis of the molecular alterations without a series of tissue biopsy. It is worth underlining that in all analyzed papers, the number of enrolled TNBC cases and healthy controls did not exceed 100. Based on the above-mentioned evidence, all of the presented results should be considered carefully, and results require additional validation in larger study sets.One of the major problems for the application of miRNAs as non-invasive cancer biomarkers is their low cancer specificity. Unfortunately, miRNAs found to be related to TNBC were also detected in blood circulation of patients suffering from other cancers (Figure 2A; Supplementary Table S1). miRNAs can serve as a potentially useful clinical screening tool, and their altered expression may be an introduction to more scrupulous diagnostics that allow earlier detection and treatment of cancer.

Using the bioinformatics tool, we analyzed all of TNBC-related, circulating miRNAs in order to predict their regulatory role in molecular pathways (WikiPathways) (Supplementary Figure S1). Most of the miRNAs are involved in the management of key cellular processes. Their alteration can initiate carcinogenesis pathways, such as apoptosis, PI3K and Wnt pathways, autophagy, DNA repair, cell differentiation, or immune responses. These findings seem to confirm the implementation of altered expression of miRNA into the development of unique molecular phenotypes of TNBC.Examples of validated targets for circulating miRNAs are presented in Table 1. Additionally, we created an miRNA–mRNA interaction model (miRNet2.0) to assess target genes for blood miRNAs and to summarize Gene Ontology (GO) and KEGG pathway enrichment analysis for the TNBC-related miRNAs (Figure 2B). The top KEGG terms for miRNAs related to TNBC were as follows: cell cycle (1.54 × 10^−8^), pathways in cancer (3.94 × 10^−8^), p53 signaling (4.19 × 10^−7^), ErbB signaling (0.0002), and Wnt signaling (0.0013).

### 2.2. lncRNA

Circulating lncRNAs demonstrate similar diagnostic accuracy as blood miRNAs, and their combination into diagnostic signatures improves test sensitivity and specificity (Table 1) [66,67,68,69,70,71,72]. In the first reported study, Liu et al., based on MA and qRT-PCR analysis, selected the three following lncRNAs, ANRIL, HIF1A-AS2, and UCA1, as promising markers for TNBC detection (AUC range of 0.827–0.840) [66]. ANRIL was also confirmed as a TNBC biomarker in another study and its diagnostic accuracy was 0.962 [67]. In the in vitro experiments, the above-mentioned ncRNA was implicated in tumor progression, cell migration, and metastases formation [66,67]. Du et al., using three blood lncRNA signatures (ANRIL, SOX2OT, ANRASSF1) were able to distinguish healthy individuals from TNBC cases with a high diagnostic accuracy of 0.990. Authors found that the expression of plasma ANRIL, SOX2OT, and ANRASSF1 was in accordance with their tissue expression [67]. It proves the potential utility of liquid biopsy as a reliable and minimally invasive tool for lncRNA detection. Interestingly, Zhang et al., based on the serum TINCR expression, distinguished between BC histological subtypes. TINCR demonstrated a significantly higher expression in TNBC individuals and distinguished TNBC from other BC subtypes with an AUC of 0.868 [72]. Unfortunately, the above-mentioned lncRNAs were also found to be deregulated in other human cancers, suggesting their low cancerspecificity [73,74]. Using bioinformatics tools, we selected top KEGG and GO terms for the circulating lncRNAs. The top KEGG terms for circulating lncRNAs were the p53 signaling pathway (1.9 × 10^−8^), small cell lung cancer (1.3 × 10^−9^), and melanoma (7.5 × 10^−7^), and for the breast cancer pathway, FDR was 8 × 10^−6^ (Figure 2C). The GO terms are summarized in Supplementary Figure S2. The top genes targeted by lncRNAs were TIA1, DDX3X, QKI, LARP7, CDKN1A, KLF2, and the CDK family, and the top miRNAs were1, 7, 10a, 10b, 31, 98, 122, 222, and 335 (LncSEA, Diana tools). Regarding the role of lncRNAs in the development of individual TNBC phenotype, these were most significantly involved in cell apoptosis (2.19 × 10^−10^), migration (7.4 × 10^−10^), and proliferation (4.25 × 10^−9^). Disease-related predicted analysis (Lnc2Cancer2.0 and MNDR2.0 tools) suggested their key involvement in BC-related carcinogenesis (4.59 × 10^−18^ and 6.21 × 10^−16^, respectively).

### 2.3. circRNAs

circRNAs are the most recently discovered ncRNA, thus their role as tumor circulating biomarkers is limited. We found only three papers related to blood circRNAs and TNBC. Therefore, it is hard to assess their reliability and tissue-specificity. Li et el. found significantly lower expression of circ0104824 in the circulation of TNBC patients in contrast to those suffering from other BC histological subtypes. However, the expression of circRNA was higher in all BC subtypes in contrast to healthy controls (AUC = 0.849). Authors also noticed that circ0104824 was clinically correlated with tumor size, estrogen, and progesterone receptor status [75]. Two other promising circulating biomarkers of TNBC are circPSMA1 and circHIF1A. circPSMA1 is probably involved in the development of unique BC subtypes by deregulation of the miR-637/Akt1/β-catenin axis and immunosuppression. While the diagnostic accuracy of this circRNA was not assessed, authors found significant differences in its serum expression between TNBC and healthy controls [76]. circHIF1A, interacting with miRNA-149-5p and *NFIB*, promotes cell proliferation and tumor migration to distant organs. This satisfactory diagnostic accuracy was achieved by distinguishing between TNBC and healthy individuals (AUC of 0.897) [76]. Using bioinformatics, we investigated the molecular function of the blood circRNAs. The top miRNAs targeted by the afore mentioned circRNAs were miRNA-34a, 34c, 151a, 325, 448, 449a, and 1179 (miRNet2.0). Creating the interaction model, circRNA–miRNA–mRNA (miRNet2.0), we selected the top KEGG terms related to TNBC: endocrine resistance (1.9 × 10^−6^), p53 signaling pathway (3.2 × 10^−5^), cell cycle (1.1 × 10^−4^), breast cancer (1.6 × 10^−4^), and mTOR signaling (9.6 × 10^−3^). The top GO terms are summarized in Supplementary Figure S3.

Diagnostic accuracy of the discussed blood-circulating ncRNAs for the non-invasive detection of TNBC is summarized in Figure 2D. Analysis of various ncRNAs allows us to compare their diagnostic accuracy for early cancer detection, and then allows the clinical selection of the most promising molecules. The above-described studies and performed bioinformatics analysis seem to confirm that combination of different classes of ncRNAs into diagnostic tests is a prospective direction for their clinical applicability, because they can represent key molecular pathways related to TNBC phenotype.

## 3. Circulating ncRNAs for TNBC Prediction and Prognosis

The disease course can be monitored unrestrictedly and ina minimally invasive manner, thanks to the application of liquid biopsy. On the one hand, it is believed that the level of expression of circulating ncRNAs reflects the tumor aggressiveness affecting patients’ survival. On the other hand, changes in ncRNA expression after the therapy can be a valuable precursor of tumor recurrence. In the Table 2 we summarized the recent studies analyzing the utility of circulating ncRNAs for TNBC prediction and prognosis [57,70,78,79,80,81,82,83,84].

### 3.1. miRNAs

Prognostic and predictive value of miRNAs for TNBC has been proven by numerous studies analyzing tissue expression of the molecules [86,87,88]. These promising findings encouraged the investigation of miRNAs as possible tumor-circulating biomarkers. Similar to the diagnostic approach, the diagnostic signatures involving a few miRNAs represent more reliable results. Constructing the four miRNAs’ blood signature, Sahlberg et al. were able to select TNBC patients at a higher risk of early death incidence (HR = 3.60). Interestingly, the same prognostic signature demonstrated the utility for both the risk of disease relapse (HR = 3.79) and discrimination between patients who relapsed and non-recurrent individuals (AUC = 0.810) [78]. In the other study, the higher diagnostic accuracy for the discrimination between TNBC patients with recurrent disease and non-recurrent cases was achieved by the application of a seven miRNA signature (AUC = 0.943) [79]. However, miRNAs can mediate the response to chemotherapy in TNBC [89,90]. Until now, only one study investigated blood miRNAs as a predictive factor for chemotherapy response. Shao et al. noticed that the two plasma miRNAs, 200a and 210, can serve as predictors for docetaxel-treated TNBC patients. First, they correlated clinical response to chemotherapy with miRNA expression. Both miRNA-200a and 210 were upregulated in non-responders (progressive disease, PD) when compared to responders group (stable disease or partial remission, SD and PR). The diagnostic accuracy measured by AUC for patients’ distinguishment was 0.866 and 0.812 respectively. miRNA-200a (OR = 0.041) and miRNA-210 (OR = 0.062) were considered as independent factors for docetaxel-based therapy in TNBC [81]. Some of the described miRNAs were also previously selected as TNBC diagnostic biomarkers (miRNA-21, 210, and 376c). We introduced all of these blood-circulating miRNAs to bioinformatics analysis to assess their role in the development of TNBC phenotype (mirPath v3.0). Interestingly, most of the miRNAs were revealed to be involved in molecular pathways crucial for BC development, includingthe Hippo signaling pathway, proteoglycans in cancer, and fatty acid metabolism and synthesis (Supplementary Figure S4).

### 3.2. lncRNAs

**Figure 3:**
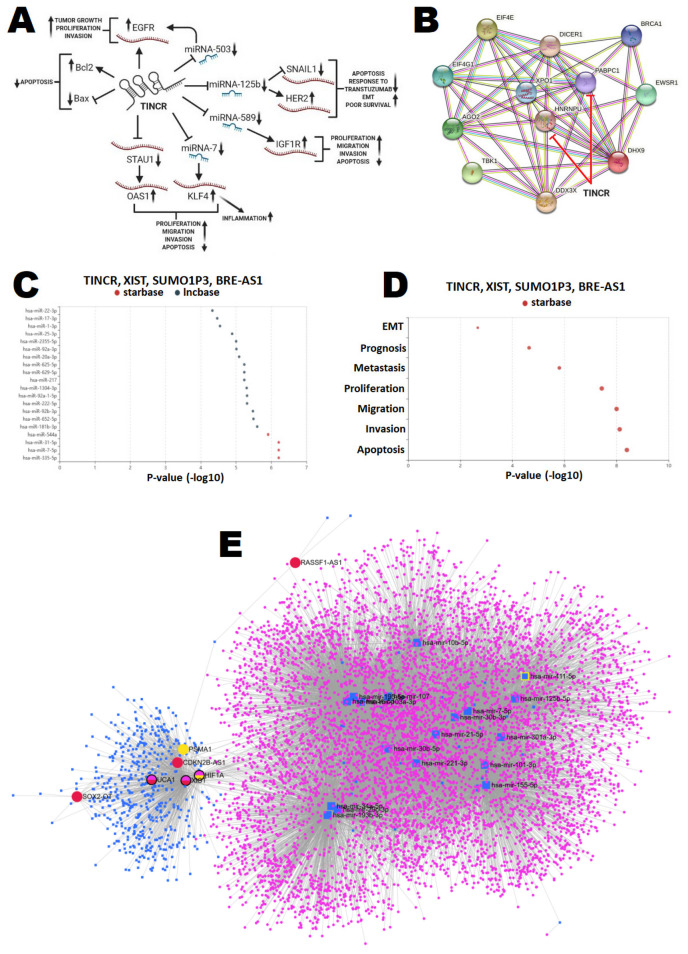
TINCR regulatory network predisposing development of unique TNBC phenotype (**A**), and bioinformatics analysis of studied ncRNAs: (**B**)—protein–protein interaction network regulated by examined lncRNAs, (**C**)—miRNA targets for the lncRNAs, (**D**)—role of the lncRNAs for establishment of aggressive phenotype of TNBC, (**E**)—ncRNAs’ interaction network among all discussed miRNAs, lncRNAs, and circRNAs.

Data regarding the utility of lncRNAs for prediction and prognosis is still limited and only few papers have focused on this idea [70,83,84,85]. Among the lncRNAs, the TINCR seems to be the most promising TNBC-related lncRNA, serving as a diagnostic and prognostic biomarker. Thorough analysis of cellular pathways mediated by TINCR seems to confirm its key function in the development of unique BC subtype (Figure 3A) [91,92].

First, TINCR enhances EGFR expression and downstream signaling via regulation of the STAT3–TINCR–EGFR pathway, and acts as a competing endogenous RNA to upregulate EGFR expression by sponging miRNA-503, resulting in tumor growth, proliferation, and migration. In vitro and in vivo experiments confirmed that TINCR knockdown suppresses BC aggressiveness [93]. TINCR targets STAU1, leading to its silencing and resulting in reduced OAS1 stability, which enhances uncontrolled cell proliferation and migration. A similar unfavorable phenotype of BC is observed under the sponging of miRNA-7 by TINCR. Downregulation of miRNA-7 leads to overexpression of KLF4, causing tumor progression and an enhanced inflammatory response in the tumor environment [94,95]. The aggressive phenotype of TNBC could be also related to the silencing of miRNA-589 and 125b. While sponging of miRNA-125b leads to overexpression of HER2 and under-expression of SNAIL1, leading to resistance to transtuzumab-based therapy and reduced survival in cancer patients, whereas silencing of miRNA-589 accelerates expression of IGF1R, reducing cell apoptosis and stimulating proliferation and invasiveness [95,96,97]. Above-discussed data found their confirmation in the clinical study considering serum TINCR as an unfavorable biomarker related to the poor course of TNBC. Wang et al. recorded that a high expression of circulating TINCR in TNBC patients is an independent prognostic factor, resulting in a higher risk of overall survival reduction (HR = 2.54). Moreover, a higher level of TINCR was found in TNBC-relapsed patients when compared with non-recurrent individuals [83]. The other promising circulating lncRNAs that could serve as survival indicators are XIST, SUMO1P3, and BRE-AS1 [70,84,85]. The circulating lncRNAs were bioinformatically tested in order to assess their role in development of aggressive phenotype of TNBC. First, the genes regulated by TINCR were selected (DIANA, LncSEA) and transferred to a protein–protein interaction network (STRING) to build a protein–protein interaction model. Interestingly, this lncRNA regulates genes involved in the BRCA1 machinery, tightly related to BC carcinogenesis (Figure 3B). Then, the target miRNAs presumptively sponged by lncRNAs (TINCR, XIST, SUMO1P3, and BRE-AS1) were selected with the use of the LncSEA tool. Figure 3C illustrates the list of miRNAs selected for the algorithm, which represent the highest binding scores with the analyzed lncRNAs. Among selected miRNAs, the miRNA-7, 31, 181b, 335, and 544a demonstrated the highest probability scores for this action, and were considered as top miRNAs for this interaction network. Regarding the tumor phenotype, the circulating lncRNAs (TINCR, XIST, SUMO1P3, and BRE-AS1) are especially important for regulation of cell apoptosis, invasion, and migration (terms achieving the highest log *p* values) (Figure 3D). Analysis of circulating lncRNAs, such as TINCR, can improve clinical management of TNBC, because this molecule participates in the regulation of pathways involved in the development of an aggressive phenotype of TNBC. In the future, TINCR can be an attractive therapeutic target for developing treatment strategies and in the selection of patients who can eventually benefit from personalized therapy. Moreover, analysis of the set of lncRNAs (TINCR, XIST, SUMO1P3, and BRE-AS1) and their targets can improve knowledge on TNBC phenotypesfor the development of selective blockades or restoration of the molecular pathways, resulting in better therapy outcomes and prolonged patients’ survival. However, further clinical validation is required to confirm the above-mentioned suppositions.

## 4. Conclusions and Perspectives

Targeted treatment strategies for TNBC, including immunotherapy, will obviously require clinically useful biomarkers for therapy enrollment, monitoring, and prediction. ncRNAs are a promising group of biomarkers that can deal with the above-mentioned expectations, which seem to confirm recent in vitro studies made on pembrolizumab—a monoclonal antibody targeting PD-1 that was approved for immunotherapy of locally recurrent unresectable/metastatic TNBC [97]. ncRNAs regulating the PD-1/PD-L1 axis are able to change the sensitivity of cells toward pembrolizumab. For instance, miRNA-34a, 138, 200c, 424, and 570, let-7a, and lncRNAs CCAT1 and GATA3-AS1 can regulate tumor growth and proliferation through PD-L1 modulation, and thus response to immunotherapy [98,99,100]. In a clinical trial (KEYNOTE-086), Loi et al., using RNA-seq-based data, confirmed that inflammatory state signatures obtained by measuring the tissue-resident memory are associated with the response to pembrolizumab in TNBC patients [101]. Prospective analysis of the blood-circulating ncRNAs can improve the selection of patients who can benefit from immunotherapy and can be a useful tool for its monitoring.

Nevertheless, a regulatory network of miRNA–lncRNA–circRNA–mRNA interactions demonstrates complex molecular machinery, in which each independent participant seems to be crucial for proper regulation of cell cycle and biological pathways. Even in the case of deregulation of single mode in this highly expanded machinery, it can lead to the failure of the entire anti-oncogenic and tumor suppressing mechanisms. To underline the complexity of ncRNA machinery, and thus the difficulty in their analysis as TNBC biomarkers, we introduced all of the discussed ncRNAs to bioinformatics interaction analysis to create a regulatory network, as well as KEGG and GO enrichment analysis (Figure 3E and Supplementary Figure S5). Noticeably, only a few introduced ncRNA can create a complex web of connections between various molecules. In the Figure 3E, the pink cluster represents genes regulated by miRNAs enrolled to the model (the miRNAs reviewed in this paper were marked by big blue squares), the blue cluster represents lncRNAs participating in the network (red dots represent lncRNAs described in this review), and the yellow dots represent reviewed circRNAs. Summarizing, the TNBC phenotype probably results from an overlap of molecular alterations, including expression of different types of ncRNA. Despite the limitations, ncRNAs can serve as prospective, minimally invasive TNBC biomarkers, detectable with the application of liquid biopsy. However, the clinical trials enrolling a considerable number of patients should be designed in order to confirm their clinical utility.

## Figures and Tables

**Table 1:** Summary of the studies evaluating blood ncRNAs as diagnostic biomarkers of TNBC (AUC—area under the ROC, HC—healthy control) (↓,↑—low or high expression).

Sample Size (*-Plasma; #-Serum)	ncRNA Expression in TNBC (Method of Detection)	Validated Targets	Biological and/or Clinical Significance of ncRNA for TNBC	Diagnostic Accuracy (AUC)	Study
**miRNA**					
67 TNBC 90 HC *	↓:miRNA-16, 21, 199a-5p	miRNA-16: AKT3, PGK1 miRNA-199a-5p: GRP78 [41]	Warburg effect mediation, cyclin E regulation, endothelial cell migration miRNA-199a-5p is associated with tumor stage	miRNA-16: 0.798 miRNA-21: 0.874 miRNA-199a-5p: 0.884	Shin 2015 [40]
23 TNBC 85 HC #	↑: miRNA-21, 221,210	miRNA-21: PDCD4, PTEN miRNA-221: p27^Kip1^, ERα miRNA-210: RAD52, HIF-1α	Oncogenic, DNA repair, cell migration, translation inhibitors, cell proliferation Correlation with tumor grade, Ki67 expression, clinical stage, lymph node status, BMI	Combination of 3 miRNA: 1.00	Thakur 2016 [42]
36 TNBC 34 HC *	↑: miRNA-Let-7c-5p, Let-7i-5p, 7, 15, 195-5p, 489-3p ↓: miRNA-199a-3p	miRNA-7: lncRNA-XIST, RELA miRNA-15: CCNE1 miRNA-195: FASN, HMGCR, ACACA, CYP27B1 [44,45,46,47]	Cancer growth Metastasis formation Cell migration Apoptosis	Combination of 7 miRNA: 0.929	Qattan 2017 [43]
74 TNBC 12 HC *	↑: miRNA-93-3p, 105	miRNA-93-3p: SFRP1 miRNA-105: GOLIM4 [49]	Promotes stemness, chemoresistance, and metastasis in TNBC Correlation with distant metastases	miRNA-93-3p: 0.657 miRNA-105: 0.928 Panel of 2: 0.939	Li 2017 [48]
31 TNBC 34 HC	↑: miRNA-126-5p, 126-3p, 144-5p, 144-3p, 301a-3p, 101-3p ↓: miRNA-664b-5p	miRNA-101: CXCR7 miRNA-126: ADAM9, RGS3 miRNA-144: PTEN miRNA-301a: ESR1 miRNA-664b: BRIP1 [51,52,53,54,55,56]	Oncogenic or tumor-suppressive regulators Cell proliferation, migration, and tumor growth Estrogen signaling pathway	Combination of 7 miRNA: 0.814	Kahraman 2018 [50]
24 TNBC 28 HC *	↑: miRNA-125b, 193b, 200b, 489	miRNA-125b: ARID3B miRNA-193b: DDAH1 miRNA-200b: VEGF-A, RARA miRNA-489: SHP2, HER2 [58,59,60]	Tumor invasion and metastasis, cell migration, angiogenesis MAPK signaling	miRNA-125b: 0.973 miRNA-193b: 0.914 miRNA-200b: 0.877 miRNA-489: 0.994	Braicu 2018 [57]
37 TNBC 34 HC *	↑: miRNA-10b, 17a, 155, 376c	miRNA-10b: HOXD4, KLF4 miRNA-17a: TIMP2, TIMP3 miRNA-155: SOCS1, Smad2, FGF, E2F miRNA-376c: RAB2A [62,63,64]	DNA repair, cell cycle procession Metastasis formation, tumor aggressiveness Correlation with tumor stage, size, lymph node status and metastasis	miRNA-10b: 0.773 miRNA-17a: 0.657 miRNA-155: 0.847 miRNA-376c: 0.866	Shaheen 2019 [61]
13 TNBC 83 HC *	↑miRNA-30b-5p	miRNA-30b-5p: CDH11, ITGA5, ITGB3	Enrichment in Wnt and p53 signaling Apoptosis Correlation with lymph node status and distant metastases	0.720	Adam-Artigues 2021 [65]
**lncRNA**					
25 TNBC 40 HC #	↑: ANRIL, HIF1A-AS2, UCA1	UCA1-miRNA-143	Invasiveness of tumor cells Activation of Wnt/β-catenin signaling Tumor progression and metastasis Correlation with lymph node status and tumor size	lncRNA-ANRIL: 0.830 lncRNA-HIF1A-AS2: 0.827 lncRNA-UCA1: 0.849	Liu 2017 [66]
100 TNBC 50 HC *	↑: ANRIL, SOX2OT, ANRASSF1	ANRIL-miRNA-199a ANRASSF1-RASSF1A [68,69]	Tumor growth and proliferation Promotion of carcinogenesis	lncRNA-ANRIL: 0.962 lncRNA-SOX2OT: 0.852 lncRNA-ANRASSF1: 0.740 Combination of 3: 0.990	Du 2018 [67]
91 TNBC 50 HC #	↑XIST	XIST-miRNA-7 XIST-miRNA-454 [71]	Tumor aggressiveness and proliferation, metastasis formation Correlation with tumor stage	0.888	Lan 2021 [70]
50 TNBC 40 BC #	↑TINCR	TINCR-miRNA-761, 125b, 503	Tumor progression, cell growth and proliferation, apoptosis regulation	TINCR allow to distinguish TNBC from BC with AUC of 0.868	Zhang 2021 [72]
**circRNA**					
83 BC (TNBC) 49 HC *	↓circ0104824	Interaction with miRNA-140, 197, 599, 677 and 1278	Cell cycle and cell proliferation Tumor stage, grading and metastasis Correlation with tumor size, estrogen, and progesterone receptor status	AUC for total BC: 0.849 Significant difference in expression between TNBC and non-TNBC and controls	Li 2020 [75]
20 TNBC 20 HC #	↑circPSMA1	PSMA1-miRNA-637	Facilitates the tumorigenesis, metastasis, cell migration through miR-637/Akt1/β-catenin axis and immunosuppression	AUC not assessed Significant difference in expression between TNBC and controls	Yang 2021 [76]
24 TNBC 68 HC *	↑circHIF1A	circHIF1A-miRNA-149-5p Interaction with NFIB	Promotion of cell proliferation and metastasis	0.897	Chen 2021 [77]

**Table 2:** Summary of the prognostic and predictive role of blood circulating ncRNAs for TNBC (AUC—area under the ROC, CR—complete response, HR—hazard ratio, OR—odds ratio, OS—overall survival, PD—progressive disease, PR—partial response, RFS—relapse-free survival, SD—stable disease) (↓,↑—low or high expression).

ncRNA	Role	Study Findings	Study
Unfavorable: ↑ miRNA-18b, ↑ miRNA-103, ↑ miRNA-107, ↑ miRNA-652 (all ↑ considered as a high risk score signature)	Prognosis/OS	- TNBC patients with high risk score (high expression of 4 miRNA) had approximately 4 fold higher risk of OS reduction (HR = 3.60)	Sahlberg 2015 [78]
	Tumor relapse/RFS	- TNBC patients with high risk score had over 3 fold higher risk of RFS reduction (HR = 3.49) - miRNA signature showed the strongest predictive value to discriminate tumors from patients with early relapse from those without recurrence (AUC = 0.810)	
Unfavorable: ↑ miRNA-21, ↑ miRNA-194, ↑ miRNA-205, ↑ miRNA-375 ↓ miRNA-376c, ↓ miRNA-382, ↓ miRNA-411	Tumor relapse	- Signature of 7 serum miRNA allows to distinguish recurrent TNBC patients from non-recurrent TNBC individuals with diagnostic accuracy of AUC = 0.943	Huo 2016 [79]
Unfavorable: ↓ miRNA-34a, 34c	Prognosis/OS	- Low expression of miRNA-34a and miRNA-34c is associated with a higher risk of early death incidence in TNBC patients (HR = 2.06 and HR = 2.47, respectively)	Zeng 2017 [80]
Unfavorable: ↓ miRNA-29c	Prognosis/OS	- Low expression of plasma miRNA-29c is an unfavorable factor associated with reduced survival in TNBC (median survival low vs. high expression of miRNA-29c: 7.6 vs. 9.6 years)	Braicu 2018 [57]
Unfavorable: ↑ miRNA-200a ↑ miRNA-210	Chemoresistance	- The expression of miRNA-200a and miRNA-210 is significantly higher in the plasma of docetaxel-resistant cases (PD) than in the sensitive individuals (PR or SD) - miRNA-200a (OR = 0.041) and miRNA-210 (OR = 0.062) were identified as independent factors for chemotherapeutic response; plasma miRNA-200a and miRNA-210 allow distinguishing between responders and non-responders with AUC of 0.866 and 0.812, respectively	Shao 2019 [81]
Unfavorable: ↓miRNA-4448, miRNA-2392, miRNA-2467, miRNA-4800	Response to chemotherapy	- A combined signature of four miRNAs could be used to discriminate between CR and non-CR patients with TNBC with an AUC of 0.765	Sueta 2021 [82]
Unfavorable: ↑ lncRNA-TINCR	Tumor relapse/RFS	- High expression of serum TINCR is related to higher rate of the disease relapse - Patients in the high serum TINCR group had poorer RFS than those in the low serum TINCR group	Wang 2020 [83]
	Prognosis/OS	- High serum expression of TINCR is associated with 2.5fold higher risk of OS reduction in TNBC patients (HR = 2.54)	
Unfavorable: ↑ miRNA-21 ↓ lncRNA-BRE-AS1	Prognosis/OS	- Patients with low plasma expression of BRE-AS1 and high expression levels of miRNA-21 showed significantly lower OS rates	Gao 2021 [84]
Unfavorable: ↑ lncRNA-XIST	Tumor relapse	- Expression of XIST in serum exosomes is higher in serum of recurrent TNBC patients than in non-recurrent individuals	Lan 2021 [70]
	Prognosis/OS	- High expression of XIST in serum exosomes is associated with reduced survival in TNBC patients (HR = 3.54)	
Unfavorable: ↑ lncRNA-SUMO1P3	Prognosis/OS	- High serum SUMO1P3 expression is independent and unfavorable prognostic factor related to poor OS in TNBC (HR = 1.97)	Na-Er 2021 [85]
	Response to chemotherapy	- No significant difference in serum SUMO1P3 was found between the pre-treated and post-treated blood samples for the chemoresistant cases. Serum SUMO1P3 levels decreased in the chemosensitive cases following the chemotherapy	

## Supplementary Materials

The following supporting information can be downloaded at: https://www.mdpi.com/article/10.3390/cancers14030803/s1, Figure S1: Heatmap of circulating miRNAs demonstrating their importance for the particular molcular pathways; Figure S2: GO enrichment analysis for circulating lncRNAs; Figure S3: GO enrichment analysis for circulating circRNAs; Figure S4: Clustering analysis of the expression of circulating miRNAs for the molecular processes related to the development of TNBC; Figure S5: GO and KEGG enrichment analysis for the regulatory network of studied ncRNAs; Table S1: The studied blood circulating miRNAs as diagnostic biomarkers of TNBC and their utility for the management of the different human cancers [102,103,104,105,106,107,108,109,110,111,112,113,114,115,116,117,118,119,120,121,122,123,124,125,126,127,128,129,130,131,132,133,134,135,136,137,138,139,140,141,142,143,144,145,146,147,148,149,150,151,152,153,154,155,156,157,158,159,160,161,162,163,164,165,166,167,168,169,170,171].
