# Supplementary material for: Blood Circulating Non-Coding RNAs for the Clinical Management of Triple-Negative Breast Cancer
Source: Cancers (Basel). 2022 Feb 4;14(3):803. doi: 10.3390/cancers14030803 (PMC8833777; doi:10.3390/cancers14030803)
Supplement: Supplementary file 1 [file cancers-14-00803-s001.zip › cancers-1585619-supplementary.pdf]

Table S1. The studied blood circulating miRNAs as diagnostic biomarkers of TNBC and their utility for the management of the different human cancers.

miRNA	Cancer	Expression in cancer	sample	role	Reference
**Let-7c-5p**	Papillary thyroid cancer	down	plasma	diagnosis	[1]
	Bladder cancer	up	urine	prediction	[2]
	Lung cancer	down	plasma	diagnosis	[3]
	Pancreatic cancer	down	plasma	diagnosis	[4]
	Gastric cancer	up	serum	diagnosis	[5]
**Let-7i-5p**	Papillary thyroid cancer	down	plasma	diagnosis	[1]
	Pancreatic cancer	down	plasma	diagnosis	[4]
	Gastric cancer	up	serum	diagnosis	[5]
	Ovarian cancer	down	serum	diagnosis	[6]
**miRNA-7**	Ovarian cancer	up	serum	Diagnosis prognosis	[7]
	Lung cancer	up	serum	response to chemotherapy	[8]
**miRNA-10b**	Lung cancer	up	serum	diagnosis	[9]
	Esophaegal cancer	up	serum	diagnosis	[10]
	Hepatocellular carcinoma	up	serum	response to sorafenib	[11]
**miRNA-15**	Bladder cancer	up	plasma	diagnosis	[12]
	SCC of lung	down	plasma	diagnosis	[13]
	Hepatocellular carcinoma	up	serum	diagnosis	[14]
**miRNA-16**	Lung cancer	up	plasma	diagnosis	[15]
	Gastric cancer	down	plasma	diagnosis disease progression	[16]
	Hepatocellular carcinoma	down	plasma	diagnosis	[17]
**miRNA-17a**	Colorectal cancer	down	plasma	prediction of recurrence	[18]
	Gastric cancer	up	plasma	diagnosis	[19]
**miRNA-21**	Lung cancer	up	plasma	Diagnosis Response to platinum	[20]
	Panceratic cancer	up	serum	diagnosis prognosis	[21]
	Colorectal cancer	up	plasma/serum	diagnosis prognosis	[22]
	Gastric cancer	up	plasma	diagnosis	[23]
	Renal cell carcinoma	up	serum	monitoring	[24]
	Ovarian cancer	up	plasma	diagnosis	[25]
**miRNA-30b**	Pancreatic cancer	down	serum	diagnosis	[26]
	Lung cancer	up	plasma	reponse to erlotinib	[27]
**miRNA-93**	Ovarian cancer	up	serum	diagnosis	[28]

	Prostate cancer	up	serum	response to therapy	[29]
	Hepatocellular carcinoma	up	plasma	diagnosis	[30]
**miRNA-101**	Gastric cancer	down	plasma	prognosis disease progression	[31]
	Hepatocellular carcinoma	up	plasma	diagnosis	[32]
	Colorectal cancer	down	serum	diagnosis prognosis	[33]
**miRNA-105**	Lung cancer	up	plasma	diagnosis	[34]
**miRNA-125b**	Lung cancer	up	serum	diagnosis prognosis	[35]
	Hepatocellular carcinoma	low	serum	prognosis	[36]
	Ovarian cancer	up	serum	diagnosis prognosis	[37]
**miRNA-126**	Lung cancer	up	plasma	diagnosis	[38]
	Prostate cancer	up	plasma	prognosis	[29]
	Colorectal cancer	up	plasma	response to bevacizumab prognosis	[39]
**miRNA-144**	Colorectal cancer	up	plasma	diagnosis	[40]
	Gastric cancer	low	serum	prognosis	[41]
	Lung cancer	up	serum	diagnosis	[42]
**miRNA-155**	Lung cancer	up	serum/plasma	diagnosis prognosis	[43]
	Hepatocellular carcinoma	up	serum	diagnosis prognosis	[44]
	Ovarian cancer	up	serum	diagnosis	[45]
**miRNA-193b**	Esophaegal cancer	down	serum	response to chemotherapy	[46]
	Lung cancer	up	serum	diagnosis	[47]
	Colorectal cancer	down	serum	diagnosis prognosis	[48]
**miRNA-195**	Lung cancer	down	plasma	diagnosis prognosis	[49]
	Hepatocellular carcinoma	down	plasma	diagnosis disease progression	[50]
	Esophaegal cancer	down	serum	diagnosis prognosis	[51]
**miRNA-199a**	Colorectal cancer	down	serum	diagnosis disease progression	[52]
	Hepatocellular carcinoma	down	serum	treatment response	[53]
	Gastric cancer	up	plasma	diagnosis	[54]
**miRNA-200b**	Ovarian cancer	up	serum	disease progression prognosis	[55]

	Prostate cancer	up	serum	diagnosis	[56]
	Colorectal cancer	up	plasma	prognosis	[57]
**miRNA-210**	Lung cancer	up	plasma	diagnosis	[58]
	Hepatocellular carcinoma	up	serum	prognosis treatment response	[59]
	Bladder cancer	up	serum	diagnosis	[60]
	Pancreatic cancer	up	plasma	diagnosis	[61]
**miRNA-221**	Pancreatic cancer	up	plasma	diagnosis monitoring prediction	[62]
	Colorectal cancer	up	plasma	diagnosis prognosis	[63]
	Ovarian cancer	up	serum	diagnosis prognosis	[64]
**miRNA-301a**	Colorectal cancer	up	serum	diagnosis	[65]
	Renal cell carcinoma	up	plasma	disease progression	[66]
**miRNA-376c**	Gastric cancer	up	plasma	diagnosis	[67]
	Colorectal cancer	up	serum	diagnosis	[68]
**miRNA-489**	Melanoma	down	serum	prediction	[69]
**miRNA-664b**	Ovarian cancer	up	whole blood	diagnosis	[70]

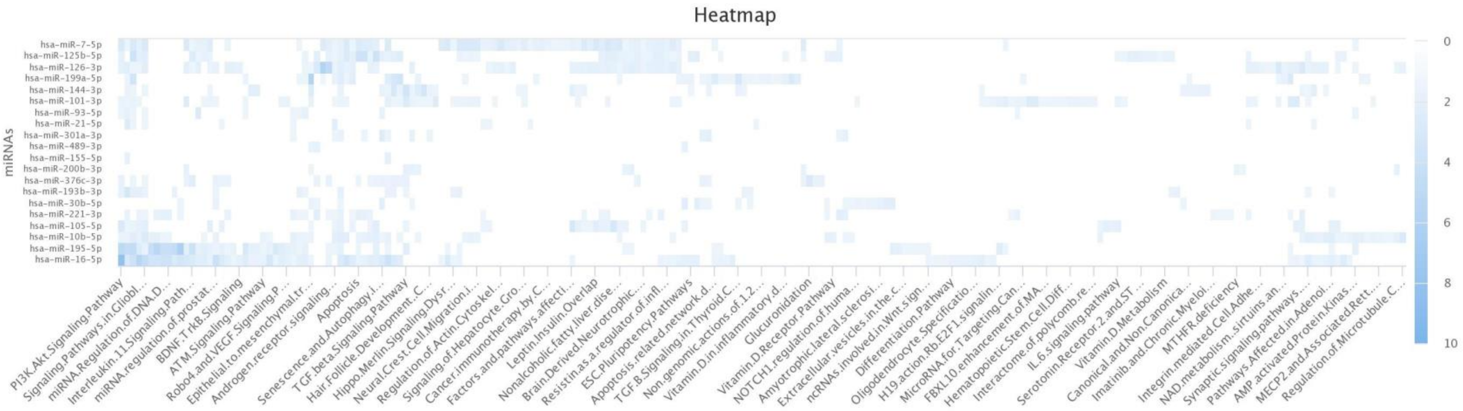

Figure S1. Heatmap of circulating miRNAs demonstrating their importance for the particular molcular pathways. (Pathways derived from WikiPathways, and heatmap created by miRPathDB 2.0).

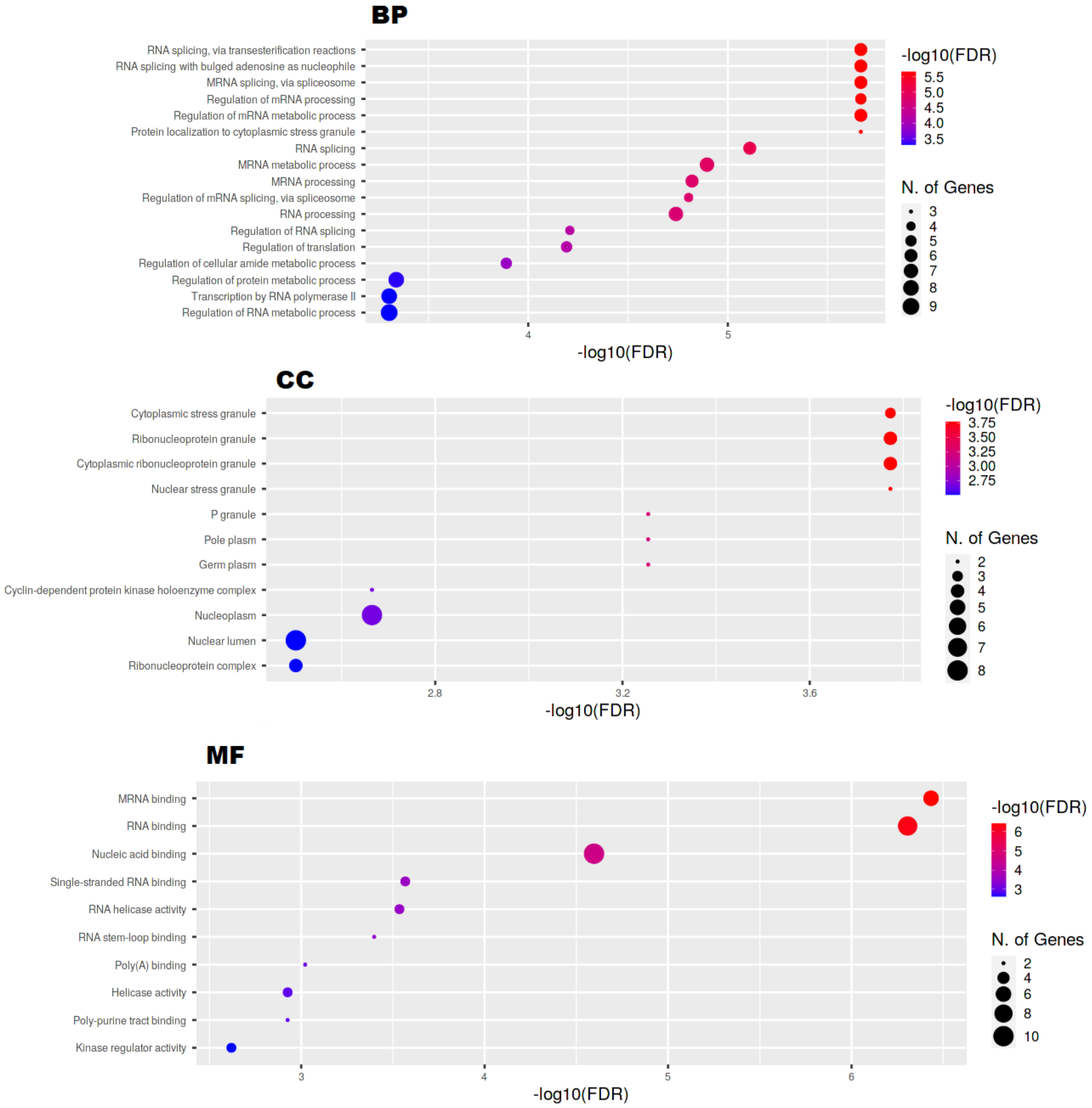

Figure S2. GO enrichment analysis for circulating lncRNAs (the top terms were summarized and plots generated using ShinyGO v0.741, String2.0 and miRNet2.0)(BP – biological process, CC – celular component, MF – molecular function).

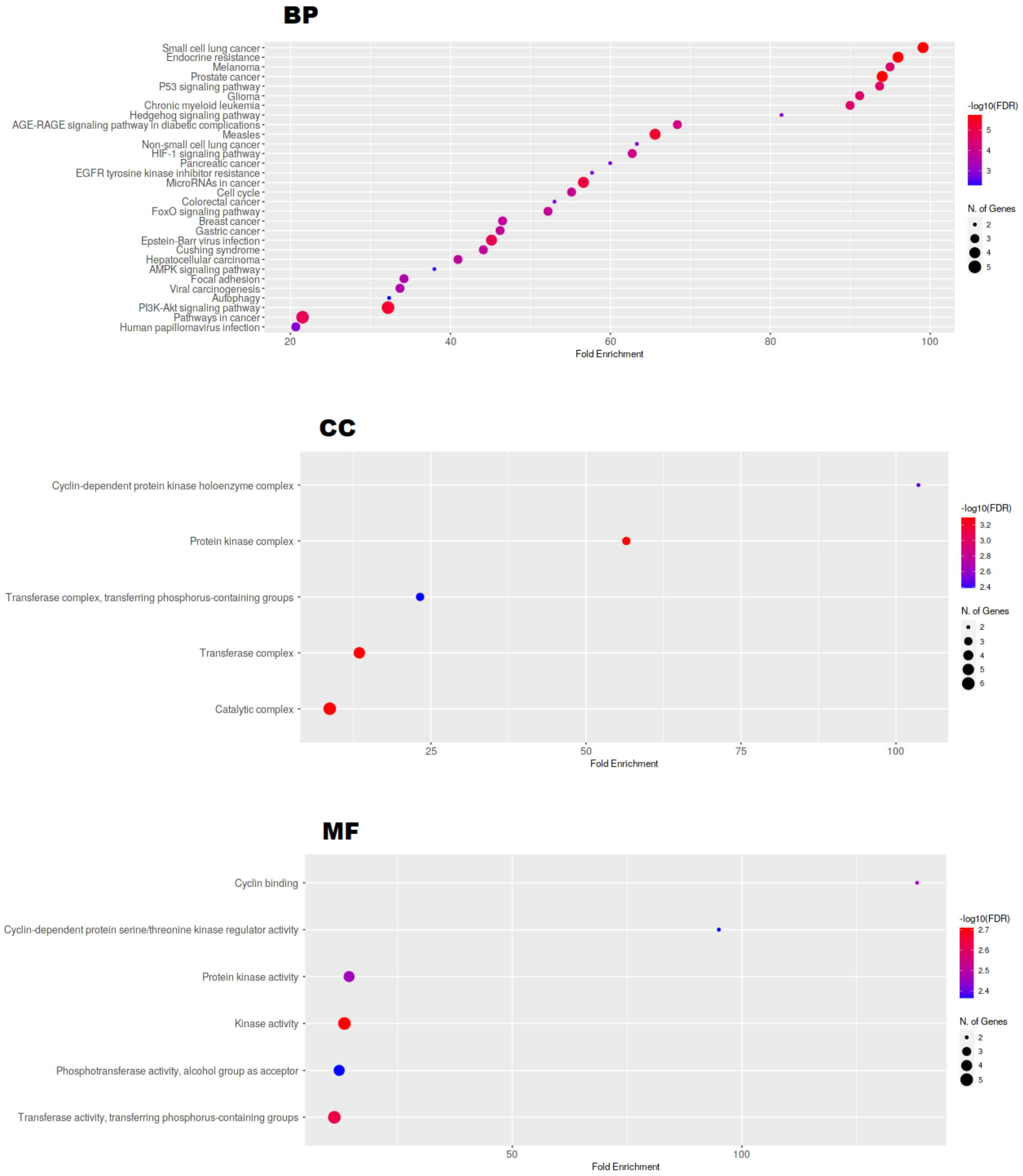

Figure S3. GO enrichment analysis for circulating circRNAs (the top terms were summarized and plots generated using ShinyGO v0.741, String2.0 and miRNet2.0)(BP – biological process, CC – celular component, MF – molecular function).

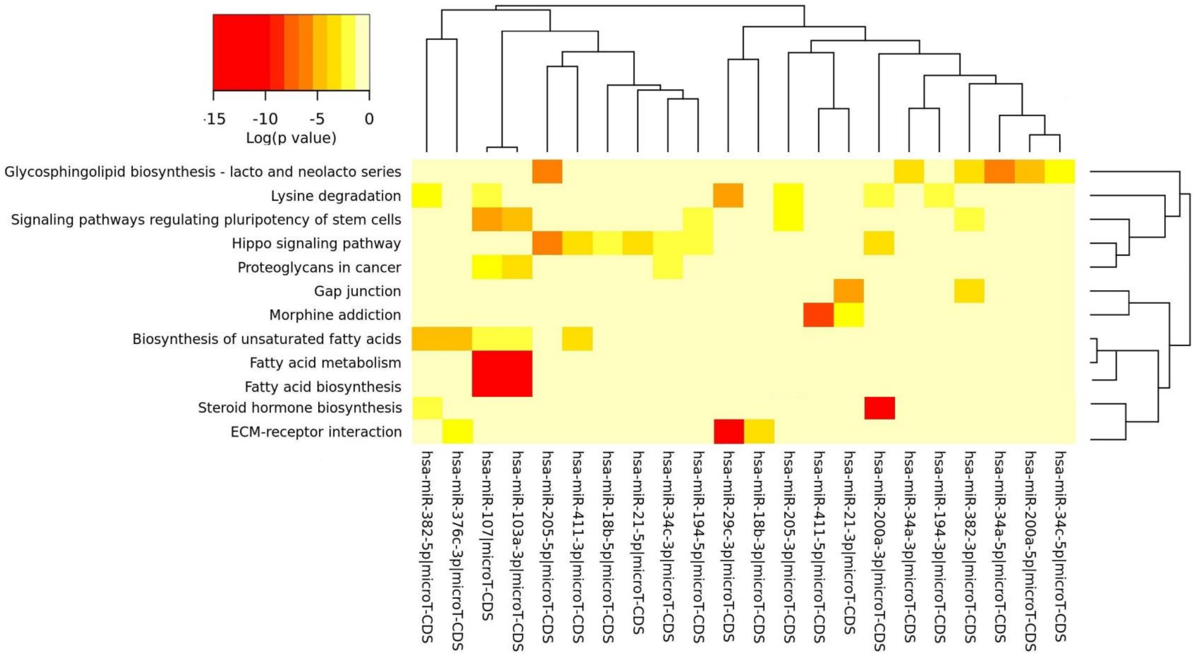

Figure S4. Clustering analysis of the expression of circulating miRNAs for the molecular processes related to the development of TNBC (DIANA tools).

KEGG

Name	Hits	Pval	adj.Pval
Pathways in cancer	196	2.18e-16	2.18e-14
Prostate cancer	69	1.82e-13	9.1e-12
Cell cycle	88	6.73e-12	2.243333e-1
Pancreatic cancer	55	3.88e-11	9.7e-10
Colorectal cancer	42	9.87e-11	1.974e-9
Neurotrophin signaling	85	1.46e-10	2.433333e-9
Chronic myeloid leuke	55	1.85e-9	2.642857e-8
HTLV-I infection	122	2.55e-9	3.1875e-8
Focal adhesion	120	2.14e-8	2.3e-7
Influenza A	72	2.3e-8	2.3e-7
Toxoplasmosis	64	3.75e-8	3.409091e-7
Renal cell carcinoma	45	7.28e-8	6.066667e-7
Small cell lung cancer	56	1.08e-7	8.307692e-7
p53 signaling pathway	49	1.62e-7	0.000001157
Glioma	47	2.46e-7	0.00000164
Acute myeloid leukem	42	4.63e-7	0.000002835
RNA transport	79	4.82e-7	0.000002835
Wnt signaling pathway	88	5.6e-7	0.000003111
Adherens junction	49	6.85e-7	0.000003605
ErbB signaling pathwa	58	8.86e-7	0.00000443
mTOR signaling pathv	34	0.000002	0.000010363

GO: BP

Name	Hits	Pval	adj.Pval
viral reproductive process	322	1.29e-13	1.29e-11
interphase of mitotic cell cycle	238	3.43e-11	1.715e-9
interphase	241	5.49e-11	1.83e-9
intracellular protein transport	399	7.87e-11	1.9675e-9
interaction with host	231	2.08e-10	4.16e-9
cell division	266	7.94e-10	1.323333e-8
protein targeting	281	2.66e-9	3.8e-8
positive regulation of cell cycle	75	5.12e-9	6.333333e-8
viral reproduction	394	5.7e-9	6.333333e-8
negative regulation of cellular protein metabolic	242	7.25e-9	7.25e-8
nuclear import	134	8.62e-9	7.836364e-8
protein transport	650	9.8e-9	8.166667e-8
establishment of protein localization	672	1.31e-8	1.007692e-7
G1/S transition of mitotic cell cycle	122	1.7e-8	1.152941e-7
nuclear transport	208	1.76e-8	1.152941e-7
nucleocytoplasmic transport	206	1.94e-8	1.152941e-7
protein import into nucleus	131	1.96e-8	1.152941e-7
protein localization	833	4.03e-8	2.238889e-7
regulation of cell cycle	425	4.66e-8	2.452632e-7

GO: MF

Name	Hits	Pval	adj.Pval
nucleotide binding	1120	1.89e-17	1.89e-15
RNA binding	484	1.98e-15	7.466667e-14
chromatin binding	198	2.24e-15	7.466667e-14
protein domain specific binding	297	2.88e-14	7.2e-13
purine nucleotide binding	862	5.95e-14	1.19e-12
purine ribonucleotide binding	859	7.32e-14	1.22e-12
ATP binding	689	2.9e-13	4.142857e-12
enzyme binding	566	8.99e-13	1.12375e-11
adenyl nucleotide binding	701	2.4e-12	2.666667e-11
adenyl ribonucleotide binding	698	4.04e-12	4.04e-11
kinase binding	218	6.11e-10	5.554545e-9
protein kinase binding	195	8.75e-9	7.291667e-8
ubiquitin-protein ligase activity	149	0.00000129	0.000009923077
protein binding transcription factor activity	279	0.00000342	0.00002442857
transcription factor binding	240	0.00000482	0.00003213333
small conjugating protein ligase activity	156	0.00000558	0.00003282353
transcription cofactor activity	156	0.00000558	0.00003282353
small conjugating protein binding	254	0.0000236	0.0001311111
single-stranded RNA binding	46	0.0000253	0.0001331579
negative regulation of transcription, DNA-deper	29	0.0000348	0.000174

GO: CC

Name	Hits	Pval	adj.Pval
cytosol	1240	4.27e-32	4.27e-30
nucleoplasm	843	7.78e-20	3.89e-18
nucleoplasm part	457	1.9e-18	6.333333e-17
chromatin	189	1.29e-15	3.225e-14
nuclear lumen	1160	6.62e-15	1.324e-13
membrane-enclosed lumen	1440	2.34e-13	3.9e-12
nucleolus	325	7.08e-13	1.011429e-11
organelle lumen	1410	1.44e-12	1.8e-11
nuclear body	163	2.62e-11	2.911111e-10
nuclear part	1360	1.24e-9	1.24e-8
nuclear chromatin	95	1.73e-9	1.572727e-8
nuclear membrane	117	2.74e-9	2.283333e-8
chromosome	361	1.05e-8	8.076923e-8
nuclear chromosome part	145	1.14e-8	8.142857e-8
nuclear chromosome	165	1.84e-8	1.226667e-7
ribonucleoprotein complex	317	2.22e-8	1.3875e-7
chromosomal part	312	2.74e-8	1.611765e-7
perinuclear region of cytoplasm	229	6.84e-8	3.8e-7
cell leading edge	143	7.44e-7	0.000003915789

Figure S5. GO and KEGG enrichment analysis for the regulatory network of studied ncRNAs. (BP – biological process; CC – cellular component; MF – molecular function).

References

1. Perdas, E.; Stawski, R.; Kaczka, K.; Zubrzycka, M. Analysis of Let-7 Family miRNA in Plasma as Potential Predictive Biomarkers of Diagnosis for Papillary Thyroid Cancer. *Diagnostics* **2020**, *10*, 130, https://doi.org/10.3390/diagnostics10030130.
2. Spagnuolo, M.; Costantini, M.; Ferriero, M.; Varmi, M.; Sperduti, I.; Regazzo, G.; Cicchillitti, L.; Mendez, A.B.D.; Cigliana, G.; Pompeo, V.; et al. Urinary expression of let-7c cluster as non-invasive tool to assess the risk of disease progression in patients with high grade non-muscle invasive bladder Cancer: a pilot study. *J. Exp. Clin. Cancer Res.* **2020**, *39*, 1–11, https://doi.org/10.1186/s13046-020-01550-w.
3. Dou, H.; Wang, Y.; Su, G.; Zhao, S. Decreased plasma let-7c and miR-152 as noninvasive biomarker for non-small-cell lung cancer. *Int. J. Clin. Exp. Med.* **2015**, *8*, 9291–9298.
4. Ali, S.; Almhanna, K.; Chen, W.; A Philip, P.; Sarkar, F.H. Differentially expressed miRNAs in the plasma may provide a molecular signature for aggressive pancreatic cancer.. *Am. J. Transl. Res.* **2010**, *3*, 28–47.
5. Liu, W.-J.; Xu, Q.; Sun, L.-P.; Dong, Q.-G.; He, C.-Y.; Yuan, Y. Expression of serum let-7c, let-7i, and let-7f microRNA with its target gene, pepsinogen C, in gastric cancer and precancerous disease. *Tumor Biol.* **2014**, *36*, 3337–3343, https://doi.org/10.1007/s13277-014-2967-9.
6. Langhe, R.; Norris, L.; Abu Saadeh, F.; Blackshields, G.; Varley, R.; Harrison, A.; Gleeson, N.; Spillane, C.; Martin, C.; O'Donnell, D.M.; et al. A novel serum microRNA panel to discriminate benign from malignant ovarian disease. *Cancer Lett.* **2015**, *356*, 628–636, https://doi.org/10.1016/j.canlet.2014.10.010.
7. Meng, X.; A Joosse, S.; Müller, V.; Trillsch, F.; Milde-Langosch, K.; Mahner, S.; Geffken, M.; Pantel, K.; Schwarzenbach, H. Diagnostic and prognostic potential of serum miR-7, miR-16, miR-25, miR-93, miR-182, miR-376a and miR-429 in ovarian cancer patients. *Br. J. Cancer* **2015**, *113*, 1358–1366, https://doi.org/10.1038/bjc.2015.340.
8. Mou, K.; Gu, W.; Gu, C.; Zhang, J.; Qwang, W.; Ren, G.; Tian, J. Relationship between miR-7 expression and treatment outcomes with gefitinib in non-small cell lung cancer. *Oncol. Lett.* **2016**, *12*, 4613–4617, https://doi.org/10.3892/ol.2016.5290.
9. Roth, C.; Kasimir-Bauer, S.; Pantel, K.; Schwarzenbach, H. Screening for circulating nucleic acids and caspase activity in the peripheral blood as potential diagnostic tools in lung cancer. *Mol. Oncol.* **2011**, *5*, 281–291, https://doi.org/10.1016/j.molonc.2011.02.002.
10. Xu, H.; Yao, Y.; Meng, F.; Qian, X.; Jiang, X.;Li, X.; Gao, Z.; Gao, L. Predictive Value of Serum miR-10b, miR-29c, and miR-205 as Promising Biomarkers in Esopha-geal Squamous Cell Carcinoma Screening. *Medicine (Baltimore)* **2015**, *94*,e1558.
11. Yoon, E.L.; Yeon, J.E.; Ko, E.; Lee, H.J.; Je, J.H.; Yoo, Y.J.; Kang, S.H.; Suh, S.J.; Kim, J.H.; Seo, Y.S.; et al. An Explorative Analysis for the Role of Serum miR-10b-3p Levels in Predicting Response to Sorafenib in Patients with Advanced Hepatocellular Carcinoma. *J. Korean Med Sci.* **2017**, *32*, 212–220, https://doi.org/10.3346/jkms.2017.32.2.212.
12. Tolle, A.; Buckendahl, L.; Jung, K. Plasma miR-15b-5p and miR-590-5p for distinguishing patients with bladder cancer from healthy individuals. *Oncol. Rep.* **2019**, *42*, 1609–1620.
13. Jin, X.; Chen, Y.; Chen, H.; Fei, S.; Chen, D.; Cai, X.; Liu, L.; Lin, B.; Su, H.; Zhao, L.; et al. Evaluation of Tumor-Derived Exosomal miRNA as Potential Diagnostic Biomarkers for Early-Stage Non–Small Cell Lung Cancer Using Next-Generation Sequencing. *Clin. Cancer Res.* **2017**, *23*, 5311–5319, https://doi.org/10.1158/1078-0432.ccr-17-0577.
14. Liu, A.M.; Yao, T.-J.; Wang, W.; Wong, K.-F.; Lee, N.P.; Fan, S.T.; Poon, R.T.P.; Gao, C.; Luk, J.M. Circulating miR-15b and miR-130b in serum as potential markers for detecting hepatocellular carcinoma: a retrospective cohort study. *BMJ Open* **2012**, *2*, e000825, https://doi.org/10.1136/bmjopen-2012-000825.
15. Sromek, M.; Glogowski, M.; Chechlinska, M.; Kulinczak, M.; Szafron, L.; Zakrzewska, K.; Owczarek, J.; Wisniewski, P.; Wlodarczyk, R.; Talarek, L.; et al. Changes in plasma miR-9, miR-16, miR-205 and miR-486 levels after non-small cell lung cancer resection. *Cell. Oncol.* **2017**, *40*, 529–536, https://doi.org/10.1007/s13402-017-0334-8.
16. Zhang, J.; Song, Y.; Zhang, C.; Zhi, X.; Fu, H.; Ma, Y.; Chen, Y.; Pan, F.; Wang, K.; Ni, J.; et al. Circulating MiR-16-5p and MiR-19b-3p as Two Novel Potential Biomarkers to Indicate Pro-gression of Gastric Cancer. *Theranostics* **2015**, *5*, 733–745
17. El-Abd, N.E.; Fawzy, N.A.; El-Sheikh, S.M.; Soliman, M.E. Circulating miRNA-122, miRNA-199a, and miRNA-16 as Biomarkers for Early Detection of Hepatocellular Carcinoma in Egyptian Patients with Chronic Hepatitis C Virus Infection. *Theranostics* **2015**, 19, 213–220.
18. Matsumura, T.; Sugimachi, K.; Iinuma, H.; Takahashi, Y.; Kurashige, J.; Sawada, G.; Ueda, M.; Uchi, R.; Ueo, H.; Takano, Y.; et al. Exosomal microRNA in serum is a novel biomarker of recurrence in human colorectal cancer. *Br. J. Cancer* **2015**, *113*, 275–281, https://doi.org/10.1038/bjc.2015.201.
19. Xu, W.; Wang, M.; Gu, H.; Wang, S.; Qian, H.; Zhu, W.; Zhang, L.; Zhao, C.; Tao, Y. Circulating miR-17-5p and miR-20a: Molecular markers for gastric cancer. *Mol. Med. Rep.* **2012**, *5*, 1514–1520, https://doi.org/10.3892/mmr.2012.828.
20. Wei, J.; Gao, W.; Zhu, C.j. Identification of plasma microRNA-21 as a biomarker for early detection and chemosensitivity of non–small cell lung cancer. *Chin. J. Cancer* **2011**, *30*, 407–414.
21. Qu, K.; Zhang X.; Lin, T. Circulating miRNA-21-5p as a diagnostic biomarker for pancreatic cancer: evidence from com-prehensive miRNA expression profiling analysis and clinical validation. *Scientific Reports* **2017**, 7, 1692.
22. Peng, Q.; Zhang, X.; Min, M.; Zou, L. The clinical role of microRNA-21 as a promising biomarker in the diagnosis and prognosis of colorectal cancer: a systematic review and meta-analysis. *Oncotarget.* **2017**, *8*, 44893–44909.
23. Nekouian, R.; Emami, S.S.; Akbari, A.; Faraji, A.; Abbasi, V.; Agah, S. Evaluation of circulating miR-21 and miR-222 as diagnostic biomarkers for gastric cancer. *J. Cancer Res. Ther.* **2018**, *15*, 115–119, https://doi.org/10.4103/jcrt.JCRT_592_17.
24. Tusong, H.; Maolakuerban, N.; Guan, J. Functional analysis of serum microRNAs miR-21 and miR-106a in renal cell car-cinoma. *Cancer Biomark.* **2017**, *18*,79–85.

25. Kartika, A.I.; Chasanah, S.N.; Fitriawan, A.S.; Tanjung, D.S.; Trirahmanto, A.; Pradjatmo, H.; Aryandono, T.; Haryana, S.M. MicroRNA-21 as a biomarker for ovarian cancer detection. *Indones. J. Biotechnol.* **2018**, *23*, 35–39, https://doi.org/10.22146/ijbiotech.35692.
26. Khan, I.A.; Rashid, S.; Singh, N. Panel of serum miRNAs as potential non-invasive biomarkers for pancreatic ductal adenocarcinoma. *Scientific Reports* **2021**, *11*, 2824.
27. Hojbjerg, J.A.; Ebert, E.B.F.; Clement, M.S.; Winther-Larsen, A.; Meldgaard, P.; Sorensen, B. Circulating miR-30b and miR-30c predict erlotinib response in EGFR-mutated non-small cell lung cancer patients. *Lung Cancer* **2019**, *135*, 92–96, https://doi.org/10.1016/j.lungcan.2019.07.005.
28. Zhang, H.; Xu, S.; Liu, X. MicroRNA profiling of plasma exosomes from patients with ovarian cancer using high-throughput sequencing. *Oncol. Lett.* **2019**, *17*, 5601–5607, https://doi.org/10.3892/ol.2019.10220.
29. Zedan, A.H.; Hansen, T.; Assenholt, J.; Pleckaitis, M.; Madsen, J.S.; Osther, P.J.S. microRNA expression in tumour tissue and plasma in patients with newly diagnosed metastatic prostate cancer. *Tumor Biol.* **2018**, *40*, https://doi.org/10.1177/1010428318775864.
30. Zhou, G.; Zeng, Y.; Luo, Y.; Guo, S.; Bao, L.; Zhang, Q. Urine miR-93-5p is a promising biomarker for early detection of HBV-related hepatocellular carcinoma. *Eur. J. Surg. Oncol. (EJSO)* **2021**, *48*, 95–102, https://doi.org/10.1016/j.ejso.2021.06.015.
31. Imamura, T.; Komatsu, S.; Ichikawa, D.; Miyamae, M.; Okajima, W.; Ohashi, T.; Kiuchi, J.; Nishibeppu, K.; Kosuga, T.; Konishi, H.; et al. Low plasma levels of miR-101 are associated with tumor progression in gastric cancer. *Oncotarget* **2017**, *8*, 106538–106550, https://doi.org/10.18632/oncotarget.20860.
32. Moshiri, F.; Salvi, A.; Gramantieri, L.; Sangiovanni, A.; Guerriero, P.; De Petro, G.; Bassi, C.; Lupini, L.; Sattari, A.; Cheung, D.; et al. Circulating miR-106b-3p, miR-101-3p and miR-1246 as diagnostic biomarkers of hepatocellular carcinoma. *Oncotarget* **2018**, *9*, 15350–15364, https://doi.org/10.18632/oncotarget.24601.
33. He, D.; Yue, Z.; Li, G.; Chen, L.; Feng, H.; Sun, J. Low Serum Levels of miR-101 Are Associated with Poor Prognosis of Colorectal Cancer Patients After Curative Resection. *Med Sci. Monit.* **2018**, *24*, 7475–7481, https://doi.org/10.12659/MSM.909768.
34. Dong, X.; Chang, M.; Song, X. Plasma miR-1247-5p, miR-301b-3p and miR-105-5p as potential biomarkers for early diag-nosis of non-small cell lung cancer. *Thorac. Cancer* **2021**, *12*, 539–548.
35. Cui, E.; Li, H..; Hua, F. Serum microRNA 125b as a diagnostic or prognostic biomarker for advanced NSCLC patients re-ceiving cisplatin-based chemotherapy. *Acta. Pharmacol. Sin.* **2013** 34, 309–313.
36. Liu, W.; Hu, J.; Zhou, K.; Chen, F.; Wang, Z.; Liao, B.; Dai, Z.; Cao, Y.; Fan, J.; Zhou, J. Serum exosomal miR-125b is a novel prognostic marker for hepatocellular carcinoma. *OncoTargets Ther.* **2017**, *ume 10*, 3843–3851, https://doi.org/10.2147/ott.s140062.
37. Zuberi, M.; Khan, I.; Mir, R.; Gandhi, G.; Ray, P.C.; Saxena, A. Utility of Serum miR-125b as a Diagnostic and Prognostic Indicator and Its Alliance with a Panel of Tumor Suppressor Genes in Epithelial Ovarian Cancer. *PLoS ONE* **2016**, *11*, e0153902, https://doi.org/10.1371/journal.pone.0153902.
38. Leng, Q.; Lin, Y.; Jiang, F.; Lee, C.-J.; Zhan, M.; Fang, H.; Wang, Y.; Jiang, F. A plasma miRNA signature for lung cancer early detection. *Oncotarget* **2017**, *8*, 111902–111911, https://doi.org/10.18632/oncotarget.22950.
39. Hansen, T.; Carlsen, A.L.; Heegaard, N.H.H.; Sørensen, F.B.; Jakobsen, A. Changes in circulating microRNA-126 during treatment with chemotherapy and bevacizumab predicts treatment response in patients with metastatic colorectal cancer. *Br. J. Cancer* **2015**, *112*, 624–629, https://doi.org/10.1038/bjc.2014.652.
40. Tan, Y.; Lin, J.-J.; Yang, X.; Gou, D.-M.; Fu, L.; Li, F.-R.; Yu, X.-F. A panel of three plasma microRNAs for colorectal cancer diagnosis. *Cancer Epidemiology* **2019**, *60*, 67–76, https://doi.org/10.1016/j.canep.2019.01.015.
41. Liu, S.; Suo, J.; Wang, C.; Sun, X.; Wang, D.; He, L.; Zhang, Y.; Li, W. Prognostic significance of low miR-144 expression in gastric cancer. *Cancer Biomarkers* **2017**, *20*, 547–552, https://doi.org/10.3233/CBM-170351.
42. Lee, H.-Y.; Song, S.-Y.; Lim, K.-H.; Park, C.-W. 490P Serum microRNAs as potential biomarkers for lung cancer. *Ann. Oncol.* **2015**, *26*, ix148–ix150, https://doi.org/10.1093/annonc/mdv533.09.
43. Shao, C.; Yang, F.; Qin, Z. The value of miR-155 as a biomarker for the diagnosis and prognosis of lung cancer: A systemat-ic review with meta-analysis. *BMC Cancer* 2019, 19,1103.
44. Ning, S.; Liu, H.; Gao, B. miR-155, miR-96 and miR-99a as potential diagnostic and prognostic tools for the clinical man-agement of hepatocellular carcinoma. *Oncol. Lett.* **2019**, *18*, 3381–3387.
45. Saeidi, N.; Saeidi, G.; Kheirandish, K. Evaluation of Circulating miRNA146a, miRNA155 and miRNA373 as Potential Biomarkers in Ovarian Cancer Detection. *J. Mol. Genet. Medicine* **2018**, *12*, 100358.
46. Chan, C.M.; Lai, K.K.Y.; Ng, E.K.O.; Na Kiang, M.; Kwok, T.W.H.; Wang, H.K.; Chan, K.W.; Law, T.T.; Tong, D.K.; Chan, K.T.; et al. Serum microRNA-193b as a promising biomarker for prediction of chemoradiation sensitivity in esophageal squamous cell carcinoma patients. *Oncol. Lett.* **2017**, *15*, 3273–3280, https://doi.org/10.3892/ol.2017.7698.
47. Nadal, E.; Truini, A.; Nakata, A.; Lin, J.; Reddy, R.M.; Chang, A.; Ramnath, N.; Gotoh, N.; Beer, D.G.; Chen, G. A Novel Serum 4-microRNA Signature for Lung Cancer Detection. *Sci. Rep.* **2015**, *5*, 12464, https://doi.org/10.1038/srep12464.
48. Xu, J.; Zhao, J.; Zhang, R. Prognostic significance of serum miR-193b in colorectal cancer.. *Int. J. Clin. Exp. Pathol.* **2017**, *10*, 9509–9514.
49. Su, K.; Zhang, T.; Wang, Y.; Hao, G. RETRACTED ARTICLE: Diagnostic and prognostic value of plasma microRNA-195 in patients with non-small cell lung cancer. *World J. Surg. Oncol.* **2016**, *14*, 1–6, https://doi.org/10.1186/s12957-016-0980-8.
50. Chen, X., Wang, A. Clinical significance of miR-195 in hepatocellular carcinoma and its biological function in tumor progression. *Onco. Targets Ther.* **2019**, *12*, 527–534.
51. Sui, D. Expression of serum microRNA-195 in patients with esophageal cancer and its clinical significance. *Chinese J. Postgraduates Medicine* **2021**, *36*, 58–62.

52. Nonaka, R.; Nishimura, J.; Kagawa, Y.; Osawa, H.; Hasegawa, J.; Murata, K.; Okamura, S.; Ota, H.; Uemura, M.; Hata, T.; et al. Circulating miR-199a-3p as a novel serum biomarker for colorectal cancer. *Oncol. Rep.* **2014**, *32*, 2354–2358, https://doi.org/10.3892/or.2014.3515.
53. Chen, Y.; Zhao, J.; Luo, Z.; Feng, C.; Hu, P.; He, X.-F.; Li, Y. Serum microRNA-199a/b-3p as a predictive biomarker for treatment response in patients with hepatocellular carcinoma undergoing transarterial chemoembolization. *OncoTargets Ther.* **2016**, *9*, 2667–2674, https://doi.org/10.2147/OTT.S98408.
54. Li, C.; Li, J.F.; Cai, Q.; Qiu, Q.Q.; Yan, M.; Liu, B.Y.; Zhu, Z.G. MiRNA-199a-3p: A potential circulating diagnostic biomarker for early gastric cancer. *J. Surg. Oncol.* **2013**, *108*, 89–92, https://doi.org/10.1002/jso.23358.
55. Zuberi, M.; Mir, R.; Das, J. Expression of serum miR-200a, miR-200b, and miR-200c as candidate biomarkers in epithelial ovarian cancer and their association with clinicopathological features. *Clin. Transl. Oncol.* **2015**, *17*, 779–787.
56. Jin, W.; Fei, X.; Wang, X. Circulating miRNAs as Biomarkers for Prostate Cancer Diagnosis in Subjects with Benign Prostat-ic Hyperplasia. *J. Immuno. Research* **2020**, *2020*, 1–9, https://doi.org/10.1155/2020/5873056
57. Yuan, Z.; Baker, K.; Redman, M. Dynamic plasma microRNAs are biomarkers for prognosis and early detection of recur-rence in colorectal cancer. *Br. J. Cancer* **2017**, *117*, 1202–1210.
58. Wang, X.; Zhi, X.; Zhang, Y.; An, G.; Feng, G. Role of plasma MicroRNAs in the early diagnosis of non-small-cell lung cancers: A case-control study. *J. Thorac. Dis.* **2016**, *8*, 1645–1652, https://doi.org/10.21037/jtd.2016.06.21.
59. Zhan, M.; Li, Y.; Hu, B.; He, X.; Huang, J.; Zhao, Y.; Fu, S.; Lu, L. Serum MicroRNA-210 as a Predictive Biomarker for Treatment Response and Prognosis in Patients with Hepatocellular Carcinoma undergoing Transarterial Chemoembolization. *J. Vasc. Interv. Radiol.* **2014**, *25*, 1279–1287.e1, https://doi.org/10.1016/j.jvir.2014.04.013.
60. Fathy, M.; Hany, N.; Bahgat, A.; Youssef, O.; Fayyad, A.; Kotb, A.; Al-Khatib, S. Circulating miR-210 and miR-23b in bladder Cancer. *Urol. Sci.* **2021**, *32*, 64, https://doi.org/10.4103/uros.uros_112_20.
61. Daoud, A.Z.; Mulholland, E.; Cole, G.; McCarthy, H.O. MicroRNAs in Pancreatic Cancer: biomarkers, prognostic, and therapeutic modulators. *BMC Cancer* **2019**, *19*, 1–13, https://doi.org/10.1186/s12885-019-6284-y.
62. Kawaguchi, T.; Komatsu, S.; Ichikawa, D.; Morimura, R.; Tsujiura, M.; Konishi, H.; Takeshita, H.; Nagata, H.; Arita, T.; Hirajima, S.; et al. Clinical impact of circulating miR-221 in plasma of patients with pancreatic cancer. *Br. J. Cancer* **2013**, *108*, 361–369, https://doi.org/10.1038/bjc.2012.546.
63. Pu, X.-X.; Huang, G.-L.; Guo, H.-Q.; Guo, C.-C.; Li, H.; Ye, S.; Ling, S.; Jiang, L.; Tian, Y.; Lin, T.-Y. Circulating miR-221 directly amplified from plasma is a potential diagnostic and prognostic marker of colorectal cancer and is correlated with p53 expression. *J. Gastroenterol. Hepatol.* **2010**, *25*, 1674–1680, https://doi.org/10.1111/j.1440-1746.2010.06417.x.
64. Hong, F.; Li, Y.; Xu, Y.; Zhu, L. Prognostic significance of serum microRNA-221 expression in human epithelial ovarian cancer. *J. Int. Med Res.* **2013**, *41*, 64–71, https://doi.org/10.1177/0300060513475759.
65. Karimi, N.; Feizi, M.A.H.; Safaralizadeh, R.; Hashemzadeh, S.; Baradaran, B.; Shokouhi, B.; Teimourian, S. Serum overexpression of miR-301a and miR-23a in patients with colorectal cancer. *J. Chin. Med Assoc.* **2019**, *82*, 215–220, https://doi.org/10.1097/jcma.0000000000000031.
66. Dias, F; Teixeira, A.; Nogueira, I. Extracellular Vesicles Enriched in hsa-miR-301a-3p and hsa-miR-1293 Dynamics in Clear Cell Renal Cell Carcinoma Patients: Potential Biomarkers of Metastatic Disease. Cancers **2020**, *12*, 1450.
67. Huang, P.S.; Chen, C.Y.; Chen, W.T. miR-376c promotes carcinogenesis and serves as a plasma marker for gastric carcino-ma. *PLoS One* **2017**, *12*, e0177346.
68. Vychytilova-Faltejskova, P.; Radova, L.; Sachlova, M.; Kosarova, Z.; Slaba, K.; Fabian, P.; Grolich, T.; Prochazka, V.; Kala, Z.; Svoboda, M.; et al. Serum-based microRNA signatures in early diagnosis and prognosis prediction of colon cancer. *Carcinogenesis* **2016**, *37*, 941–950, https://doi.org/10.1093/carcin/bgw078.
69. Mo, H.; Guan, J.; Yuan, Z.-C.; Lin, X.; Wu, Z.-J.; Liu, B.; He, J.-L. Expression and predictive value of miR-489 and miR-21 in melanoma metastasis.. *World J. Clin. Cases* **2019**, *7*, 2930–2941, https://doi.org/10.12998/wjcc.v7.i19.2930.
70. Kurt, B.; Tuncer, S.; Odemis, D. The Aberrant Expression Levels of miR-423-5p and miR-664b-5p in Peripheral Blood of Patients With Familial and Sporadic Ovarian Carcinoma. **2021**, doi:10.21203/rs.3.rs-84141/v1
